# Plate fixation or intramedullary fixation of humeral shaft fractures – an update

**DOI:** 10.3109/17453674.2012.695677

**Published:** 2012-06-04

**Authors:** David J. Heineman, Mo Bhandari, Rudolf W. Poolman

*Sir*—In April 2010 we published a meta-analysis with the title “Plate fixation or intramedullary fixation of humeral shaft fractures” in Acta Orthopaedica (Heineman et al. 2010). This meta-analysis was updated with a letter-to-the-editor in August 2010 in Acta Orthopaedica.

Since this last update new trials have been published on this subject, therefore justifying a new update of this meta-analysis. Regarding our primary outcome, total complication rate, one new trial has been published by Singisetti and Ambedkar (2010) describing the results of a prospective, comparative study of management of acute humeral shaft fractures by antegrade interlocking nail fixation and dynamic compression plating. Another new trial was published by Li et al. in 2011. This study is a prospective designed trial to compare the effect of antegrade nail with ORIF on shoulder function and range of motion. Unfortunately this study does not mention total complication rate, the primary outcome of our meta-analysis. They do mention non-union, infection and nerve damage though, three of our secondary outcomes. We decided to add all their mentioned complications together as the total complication rate. Next to these 2 new studies we decided to include a study by Kesemenli et al. from 2003, which seems to match our in- and exclusion criteria but wasn’t included in our primary meta-analysis. Therefore we add 3 new trials in this update.

By including these three trials we could add 155 patients in total. Regarding our primary outcome total complication rate we can state that this new update shows a less significant result favoring plates compared to nails (RR 0.63, CI = 0.41–0.97, p = 0.03) (Figure). It is remarkable that the confidence interval is smaller than it was in our last update (CI = 0.30–0.91), suggesting less heterogeneity. Regarding our secondary outcomes, non-union, infection and nerve palsy, this new analysis shows no new significant differences (Table).

**Figure.**
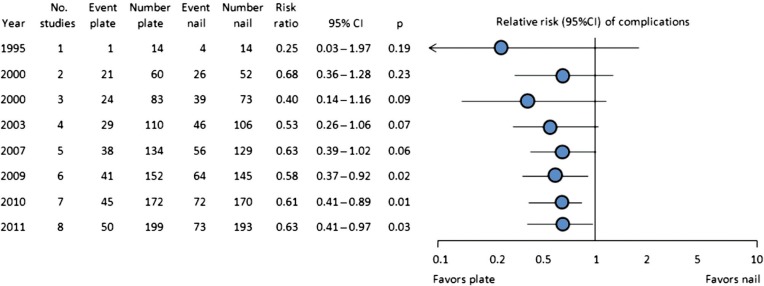
Cumulative meta-analysis of primary outcome: total complication rate.

**Table.** Secondary outcomes

Secondary outcome	Event plate	Number plate	Event IMN	Number IMN	RR	CI	p-value
Non-union	13	199	17	193	0.78	0.37–1.64	0.5
Infection	8	185	3	179	1.93	0.57–6.58	0.3
Nerve palsy	10	185	8	179	1.06	0.30–3.79	0.9

After updating our analysis we can conclude that the current literature continues to favor plates over intramedullary nails in humeral shaft fractures in the reduction of complication rates. However, the precision of our estimate is markedly improved (CI = 0.41–0.97 instead of CI = 0.30–0.91). We have to remark though that the significance is a bit less than it was in 2010 (p = 0.03 instead of p = 0.01). Regarding our secondary outcomes there still is no significant difference between nails and plates.

The weaknesses stated in our last update still remain viable: small studies with heterogeneity remain the basis of this meta-analysis. The need for a large scale RCT reporting on patient important endpoints such as complication rate and validated patient reported outcomes still remains.
